# Pathological Characteristics of a Quail Model with Hyperuricemia Combined with Hyperlipidemia

**DOI:** 10.3390/metabo15120788

**Published:** 2025-12-10

**Authors:** Shujia Liu, Xinyu Feng, Xinlong Liu, Yan Lin, Bing Zhang, Zhijian Lin, Yu Wang

**Affiliations:** School of Chinese Materia Medica, Beijing University of Chinese Medicine, Beijing 102488, China; liu2017652540@163.com (S.L.);

**Keywords:** quail, hyperuricemia, hyperlipidemia, model, high-purine high-fat

## Abstract

Objective: To explore the association between uric acid and the prevalence of hyperlipidemia via the NHANES database, a combined hyperuricemia–hyperlipidemia (HUA-HLP) quail model was subsequently established to investigate the pathological characteristics of the model. Methods: In the NHANES database, information on patients with hyperuricemia is collected, and the association between serum uric acid levels and the prevalence of hyperlipidemia is analyzed by adjusting for confounding variables. A high-purine and high-fat diet was prepared with a ratio of regular feed–yeast extract powder–lard = 15:2:3. By measuring uric acid and blood lipid levels, and observing the activities of uric acid-producing enzymes and enzymes related to lipid metabolism synthesis and decomposition, the metabolic disorder and pathological characteristics of the model were evaluated. Results: By adjusting for confounding variables, it is found that as serum uric acid levels increase, the prevalence of hyperlipidemia rises significantly. The high-purine and high-fat diet successfully induced a quail model of hyperuricemia combined with hyperlipidemia. During the first week, serum uric acid, triglyceride, total cholesterol, and low-density lipoprotein cholesterol levels were significantly elevated and remained high until the end of the experiment. Serum free fatty acid levels were significantly increased from the second week and remained at a high level. Serum high-density lipoprotein cholesterol levels were significantly reduced from the third week and remained stable thereafter. In addition, the enzymes involved in uric acid synthesis as well as those related to lipid metabolism (including synthesis and decomposition) also exhibited significant abnormalities. Conclusions: In the human body, uric acid and lipid metabolism interact with each other and exacerbate one another’s abnormalities. A high-purine and high-fat diet can induce a quail model of hyperuricemia combined with hyperlipidemia. Uric acid and lipid metabolism are simultaneously disturbed, and the activities of uric acid-producing enzymes as well as enzymes related to lipid metabolism synthesis and decomposition are also altered.

## 1. Introduction

In recent years, with the improvement of material living standards and changes in dietary patterns among the Chinese population, dietary imbalance and a high incidence of metabolic disorders have become increasingly common. Hyperuricemia (HUA) is a metabolic disease caused by disturbances in uric acid metabolism [1,2], while hyperlipidemia (HLP) is characterized by disorders of lipid metabolism [3,4,5]. Clinical reports indicate that HUA and HLP often occur together [6,7,8,9], severely disrupting various metabolic processes and increasing the metabolic burden on the body. However, there are currently no targeted therapeutic drugs available in clinical practice. Therefore, establishing an experimental animal model of HUA-HLP that mimics the course of disease development is of great significance for exploring disease mechanisms, developing new drugs, and evaluating drug efficacy.

Quails lack uricase in their bodies, which allows them to simulate the way humans handle uric acid in purine metabolism. Their dietary intake easily induces changes in lipid metabolism, which is similar to the etiology and disease course seen in clinical patients with unbalanced dietary structures.

First, this study analyzed the association between uric acid and the prevalence of hyperlipidemia based on the NHANES database. Building on human data, quails were used as experimental animals, and a combined HUA-HLP quail model was induced by a high-purine and high-fat diet to observe the pathological characteristics related to uric acid and lipid metabolism.

## 2. Materials and Methods

### 2.1. NHANES Data Analysis

#### 2.1.1. Research Object

This research is a cross-sectional analysis. The data were from the NHANES database of adult participants in six cycles from 2005 to 2016 (https://www.cdc.gov/nchs/nhanes/index.htm, accessed on 12 November 2024). The database has been approved by the Ethics Review Board of the National Center for Health Statistics of the United States, and the survey data files are published online every two years. Inclusion criteria: demographic characteristics, laboratory examination indicators, imaging examination results and questionnaire data were complete. Exclusion criteria: (1) age < 20 years old; (2) those with missing blood lipid data; (3) HUA diagnostic information missing.

#### 2.1.2. Clinical Data Collection and Grouping

In this research, gender, age, race, education level, marital status, poverty income ratio, smoking and drinking status, physical activity, hypertension, diabetes, weight, BMI and other information were collected.

Diagnostic criteria for hyperuricemia: Serum uric acid was measured as part of the standard biochemistry profile used to diagnose and treat a series of diseases. The Cel DxC 800 Synchron was used to measure the concentration of uric acid in serum. In this study, the diagnostic criteria for hyperuricemia were as follows: uric acid level was higher than 7 mg/dL in men and higher than 6 mg/dL in women [10,11,12]. A standard biochemistry test was conducted by trained laboratory technicians, and uric acid concentration was measured using a timed endpoint method. Detailed instructions about analytical methodologies, principles, and operating procedures are shown in the NHANES Laboratory Method Files.

#### 2.1.3. Hyperlipidemia Diagnostic Criteria

Hyperlipidemia was identified when any of the following criteria were met: triglycerides ≥ 150 mg/dL; total cholesterol ≥ 200 mg/dL; low-density lipoprotein ≥ 130 mg/dL; high-density lipoprotein ≤ 40 mg/dL (male); high-density lipoprotein ≤ 50 mg/dL (female); or utilization of antihyperlipidemic agents [13,14].

#### 2.1.4. Covariate Definition

Covariables contained gender, age, race, marital status, poverty, education level, drinking status, smoking status, body mass index (BMI), diabetes, hypertension.

#### 2.1.5. Statistical Analysis

This research used R software (4.3.3) for data analysis. Firstly, descriptive statistics are used for general data, continuous variables are expressed by $\bar{x}$ ± s, and categorical variables are expressed by use cases (%). The related risk factors were identified by single factor analysis. The categorical variables were tested by χ^2^ test, and the continuous variables were tested by t test or single factor logistic regression. Then it was weighted and included in the multivariate logistic regression model to analyze the relationship between uric acid and hyperlipidemia. The results were presented as odds ratio (OR) and confidence interval (CI). In R 4.3.3, the rcsplot command was used to construct a restricted cubic spline (RC) curve to fit the correlation between uric acid and hyperlipidemia, with the median as the curve reference point. *p* < 0.05 (bilateral) was considered statistically significant. Among these, continuous variables refer to abnormal blood lipid levels, i.e., the presence of abnormalities in any one of triglycerides (TG), total cholesterol (TC), low-density lipoprotein cholesterol (LDL-C), and high-density lipoprotein cholesterol (HDL-C).

### 2.2. Animal Experiments

#### 2.2.1. Animals

Twenty Defake quails, male, weighing (150 ± 10) g, were purchased from Beijing Quail King Breeding Farm, with animal quarantine certificate No. 1101195839. All quails were maintained at room temperature (23 ± 1) °C, humidity (45 ± 5)%, under 12 h light conditions, and acclimatized for 3 days. Animal ethics approval number: 2024103003-4228.

#### 2.2.2. Reagents and Instruments

Yeast extract powder (Oxoid, UK, batch number: 4448128-02);

Lard (Luoheshianghui Edible Oil Technology Co., Ltd., Luohe, China, product standard number: Q/DZSH 0024S);

Uric acid assay kit (Zhongsheng Beikong Biotechnology Co., Ltd., Beijing, China, batch number: 241442);

Triglyceride assay kit (Nanjing Jiancheng Bioengineering Institute, Nanjing, China, batch number: 20250418);

Total cholesterol assay kit (Nanjing Jiancheng Bioengineering Institute, Nanjing, China, batch number: 20250416);

Low-density lipoprotein cholesterol assay kit (Nanjing Jiancheng Bioengineering Institute, Nanjing, China, batch number: 20250320);

High-density lipoprotein cholesterol assay kit (Nanjing Jiancheng Bioengineering Institute, Nanjing, China, batch number: 20250319);

BCA protein concentration assay kit (Nanjing Jiancheng Bioengineering Institute, Nanjing, China, batch number: 20250103);

Xanthine oxidase assay kit (Nanjing Jiancheng Bioengineering Institute, Nanjing, China, batch number: 20250411);

Adenosine deaminase assay kit (Nanjing Jiancheng Bioengineering Institute, Nanjing, China, batch number: 20250410);

Non-esterified fatty acid assay kit (Addisen Biotechnology Co., Ltd., Yancheng, China, batch number: ADS20250429);

Fatty acid synthase assay kit (Addisen Biotechnology Co., Ltd., Yancheng, China, batch number: ADS20250530);

Acetyl-CoA carboxylase assay kit (Addisen Biotechnology Co., Ltd., Yancheng, China, batch number: ADS20250530);

Microplate reader (Teacan, Canton of Zurich, Switzerland, model: Sunrise);

High-speed refrigerated centrifuge (Sigma, Osterode am Harz, Germany, model: 3K15);

Constant temperature water bath (Beijing Medical Equipment Factory Co., Ltd., Beijing, China, model: HH-600);

Automatic biochemical analyzer (Jinan Bohang Biotechnology Co., Ltd., Jinan, China, model: Bokel BK-200);

Electronic balance (Shimadzu, Kyoto City, Japan, AHIMADZUB1-220H).

#### 2.2.3. Grouping and Model Establishment

Defake quails were randomly divided into a normal group and a model group according to body weight, with 10 quails in each group. The high-purine and high-fat diet was prepared by mixing regular feed, yeast extract powder, and lard at a ratio of 15:2:3, and was provided to the model group quails, who were allowed free access to drinking water. The quails in the normal group were fed and watered ad libitum. The experiment lasted for 35 consecutive days.

#### 2.2.4. Detection Indicators and Methods

##### Biochemical Index Detection

On days 7, 14, 21, 28, and 35 of modeling, blood samples were collected from the jugular vein of quails in both groups. The quails were fasted for 12 h but allowed free access to water prior to blood collection. The samples were centrifuged at 1600× *g* for 10 min to separate the serum. According to the instructions of the respective assay kits, the levels of serum uric acid (UA), triglyceride (TG), total cholesterol (TC), low-density lipoprotein cholesterol (LDL-C), high-density lipoprotein cholesterol (HDL-C), and non-esterified fatty acid (NEFA) were measured.

One day prior to blood collection, mixed fecal and urinary samples were collected from both groups. Fecal samples were fully dissolved in physiological saline, centrifuged at 1600× *g* for 10 min, and the supernatant was separated. Fecal uric acid content was measured according to the instructions of the assay kit.

##### Mechanism-Related Index Detection

According to the assay kit instructions, the activities of serum xanthine oxidase (XOD) and adenosine deaminase (ADA) were measured.

After euthanasia, the quails’ livers were dissected and homogenized. The activities of xanthine oxidase, adenosine deaminase, fatty acid synthase (FAS), lipoprotein lipase (LPL), hepatic lipase (HL), and acetyl-CoA carboxylase (ACC) in the liver homogenate were measured.

##### Pathological Tissue Section

After euthanasia, the quails’ livers and kidneys were dissected, and the sections were stained using the conventional hematoxylin-eosin (HE) staining method. Finally, observation and photography were performed under a light microscope.

#### 2.2.5. Statistical Methods

Statistical analysis of the data was performed using SPSS 27.0. Measurement data that followed a normal distribution were expressed as mean ± standard deviation. Comparison of data between the two groups was performed using the independent samples *t*-test or nonparametric tests. A corrected *p* value of less than 0.05 was considered statistically significant.

## 3. Experimental Results

### 3.1. Weighted Baseline Characteristics of Included Participants

This study included 2172 subjects, including 1012 males (46.6%) and 1160 females (53.4%), with an average age of 48.97 ± 6.69 years. A total of 379 cases of hyperuricemia were detected. The age of the hyperuricemia group was significantly lower than that of the control group (*p* < 0.001). In the hyperuricemia group, Mexican Americans accounted for 50.6%, non-Hispanic blacks accounted for 38.1%, and non-Hispanic whites accounted for 18.2%. Compared with the control group, the proportion of ‘inactivity’ in the hyperuricemia group was higher (*p* < 0.001); BMI and prevalence of hypertension in hyperuricemia group were significantly higher than those in control group (*p* = 0.001). See Table 1.

### 3.2. Association Between Hyperlipidemia and Serum Uric Acid Among US Adults in NHANES 2005–2016

The results of Logistic regression analysis showed that the prevalence of hyperlipidemia increased by 4.18 times for each unit increase in uric acid level without adjusting the covariant. After adjusting for age, gender and race, it was found that for every unit increase in uric acid level, the prevalence of hyperlipidemia increased by 3.50 times; adjusted for age, sex, race, BMI, alcohol consumption, smoking status, diabetes and hypertension, the prevalence of hyperlipidemia increased by 1.03 times for each unit of uric acid level. See Table 2.

### 3.3. The Relationship Between the Risk of Hyperlipidemia and Uric Acid Levels

**Figure 1:**
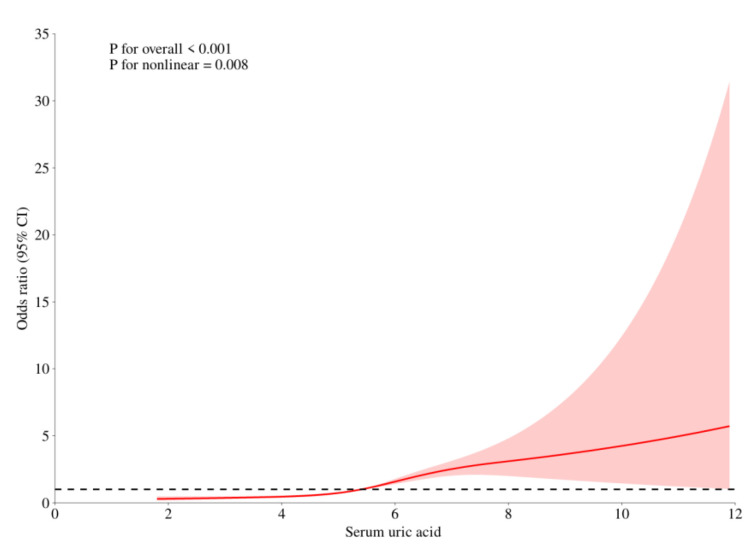
The Relationship between the Risk of Hyperlipidemia and Uric Acid Levels. (Note: The red curve represents the association trend line between serum uric acid (x-axis) and the target outcome (y-axis: Odds Ratio, i.e., OR value); the red area indicates the 95% confidence interval (95% CI) of the OR value, which means that statistically, there is a 95% probability that the true association trend falls within this area; the dashed line is the reference line where OR = 1).

RCS curve fitting was performed after adjusting covariates such as gender, age, race, BMI, drinking, smoking status, diabetes and hypertension. The results showed that there was a non-linear relationship between uric acid level and the prevalence of hyperlipidemia, and the incidence of hyperlipidemia increased more significantly in high uric acid status. See Figure 1.

### 3.4. General Condition Changes in Model Animals

**Figure 2:**
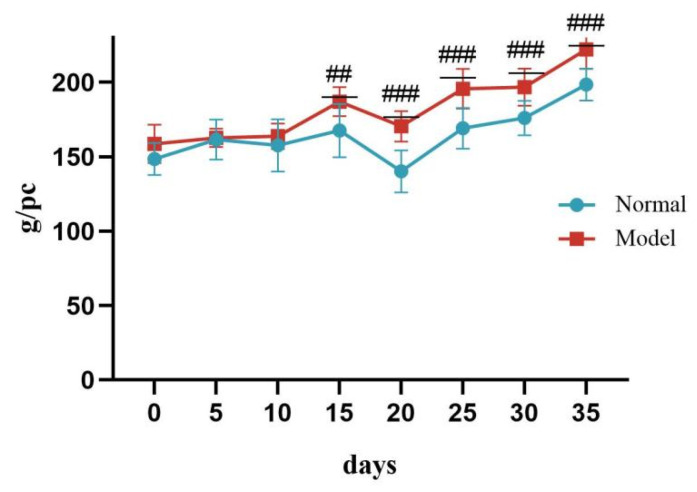
Changes in Body Weight of Quails During the Experiment. (Note: Compared with the normal group, ^##^ *p* < 0.01, ^###^ *p* < 0.001).

During days 0–10 of the experiment, there was no significant difference in body weight between the two groups of quails (*p* > 0.05). On day 15, the body weight of quails in the model group was significantly higher than that of the normal group, and continued to increase until day 35 (^##^ *p* < 0.01, ^###^ *p* < 0.001). See Table 3 and Figure 2.

**Figure 3:**
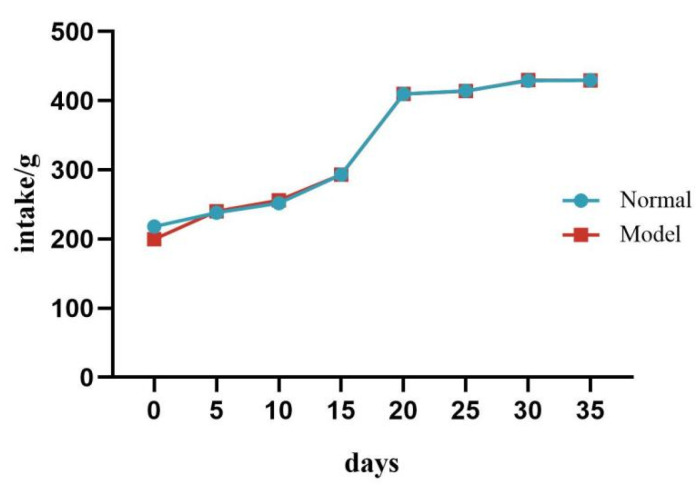
Changes in Food Intake of Quails During the Experiment.

During the experiment, there was no significant difference in food intake between the two groups of quails (*p >* 0.05). See Table 4 and Figure 3.

### 3.5. Uric Acid and Lipid Levels in Model Animals

**Figure 4:**
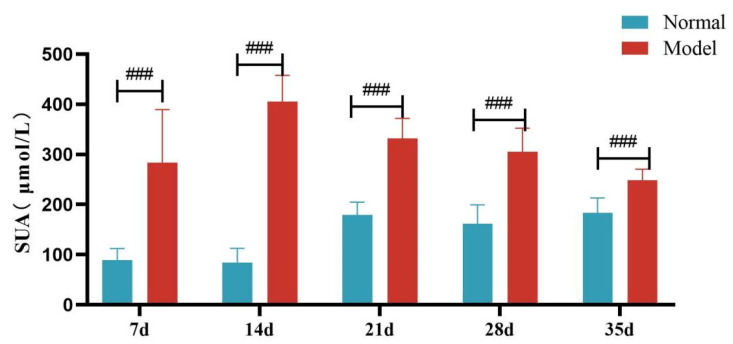
Serum Uric Acid Levels of Quails at 7, 14, 21, 28 and 35 Days. (Note: Compared with the normal group, ^###^ *p* < 0.001).

**Figure 5:**
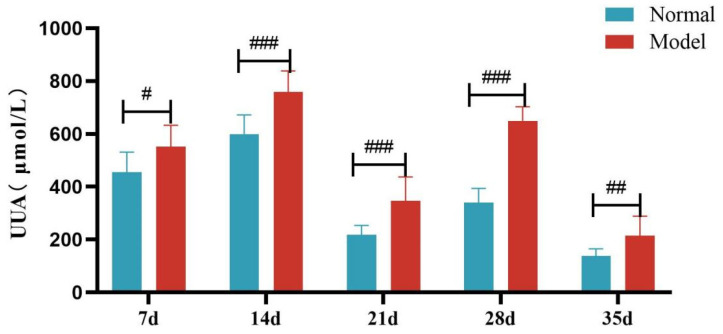
Fecal Uric Acid Levels of Quails at 7, 14, 21, 28, and 35 Days. (Note: Compared with the normal group, ^#^ *p* < 0.05, ^##^ *p* < 0.01, ^###^ *p* < 0.001).

Compared with the normal group, the serum UA level and the fecal-and-urinary output of quails in the model group were significantly or extremely significantly increased at 7, 14, 21, 28, and 35 days (*p* < 0.05, *p* < 0.01, *p* < 0.001). See Table 5 and Figure 4 and Figure 5.

**Figure 6:**
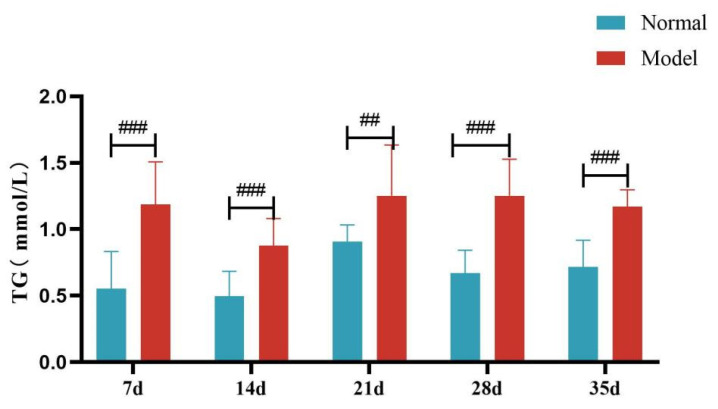
Serum TG Levels of Quails at Days 7, 14, 21, 28, and 35. (Note: Compared with the normal group, ^##^ *p* < 0.01, ^###^ *p* < 0.001).

**Figure 7:**
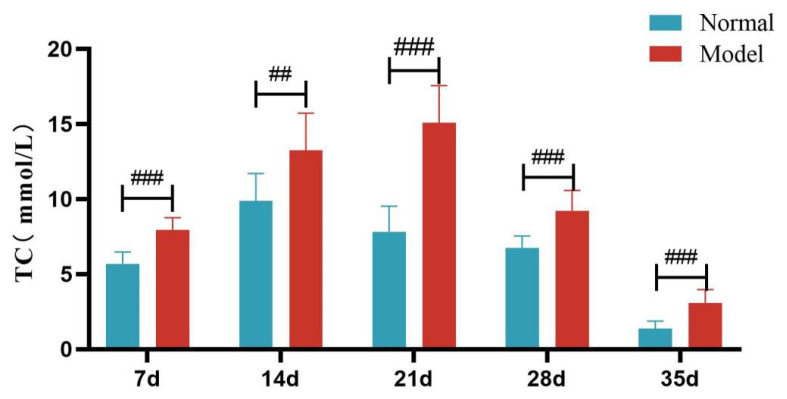
Serum TC Levels of Quails at Days 7, 14, 21, 28, and 35. (Note: Compared with the normal group, ^##^ *p* < 0.01, ^###^ *p* < 0.001).

**Figure 8:**
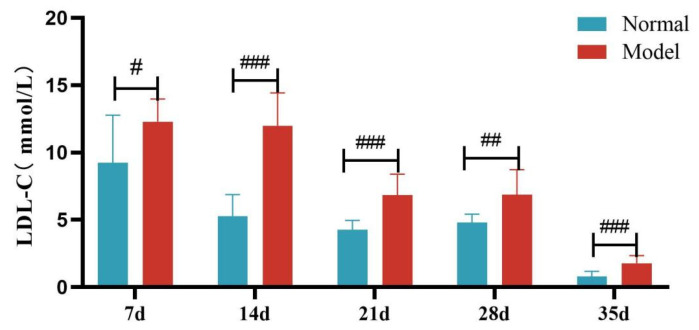
Serum LDL-C Levels of Quails at Days 7, 14, 21, 28, and 35. (Note: Compared with the normal group, ^#^ *p* < 0.05, *^##^ p <* 0.01, ^###^ *p* < 0.001).

**Figure 9:**
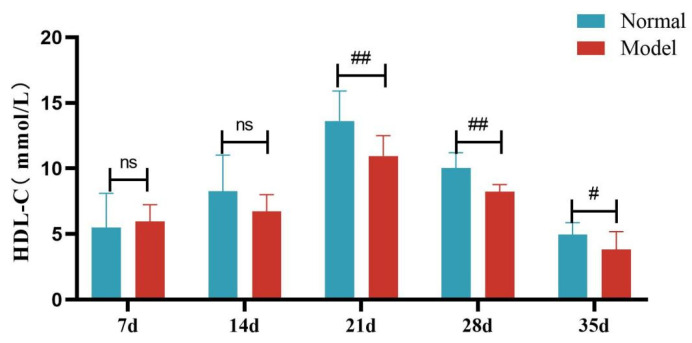
Serum HDL-C Levels of Quails at Days 7, 14, 21, 28, and 35. (Note: Compared with the normal group, ^#^ *p* < 0.05, ^##^ *p* < 0.01, ns indicates *p* > 0.05).

Compared with the normal group, TG, TC and LDL-C levels of quails in the model group were extremely significantly elevated at days 7, 14, 21, 28, and 35 (*p* < 0.05, *p* < 0.01, *p* < 0.001). There was no significant difference in the serum HDL-C levels between the two groups at days 7 and 14 (*p >* 0.05). However, at days 21, 28, and 35, serum HDL-C levels were significantly reduced in the model group (*p* < 0.05, *p* < 0.001). See Table 6 and Figure 6, Figure 7, Figure 8 and Figure 9.

**Figure 10:**
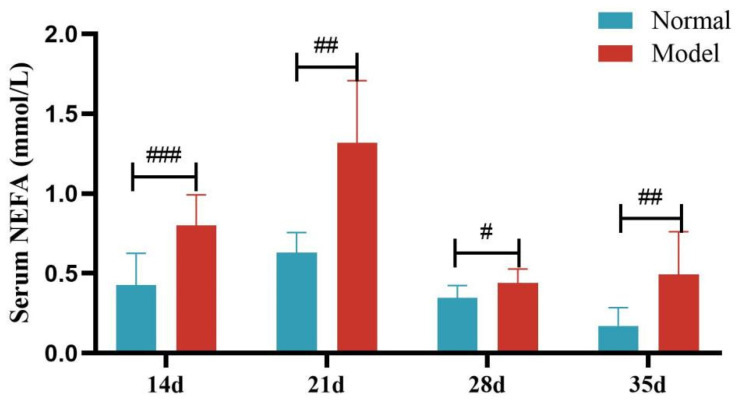
Serum FFA Levels of Quails at Days 14, 21, 28, and 35. (Note: Compared with the normal group, ^#^ *p* < 0.05, ^##^ *p* < 0.01, ^###^ *p* < 0.001).

**Figure 11:**
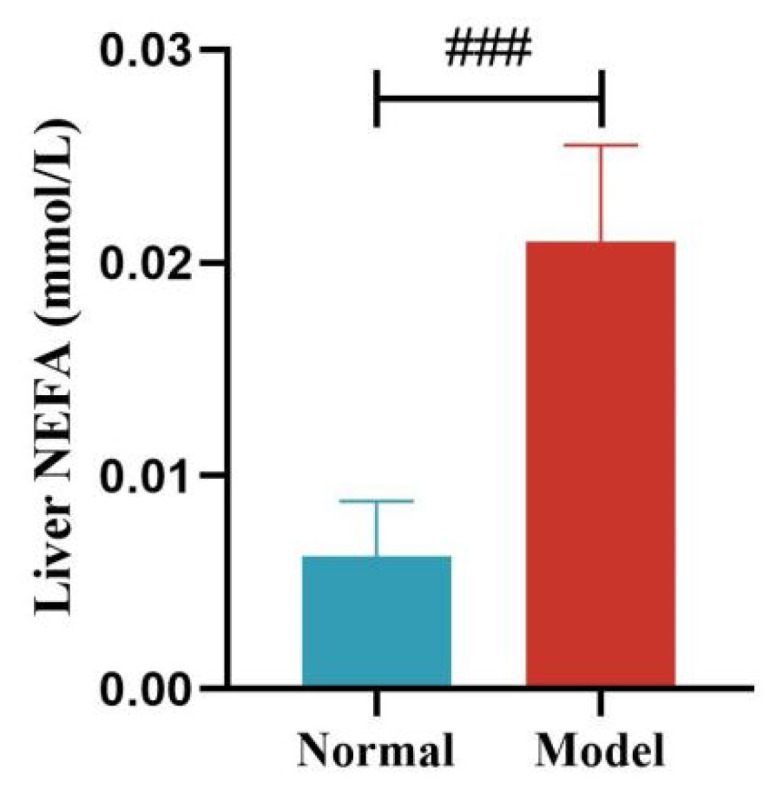
Liver NEFA Levels of Quails. (Note: Compared with the normal group, ^###^ *p* < 0.001).

Compared with the normal group, the serum NEFA levels of quails in the model group were significantly or extremely significantly increased at days 14, 21, 28, and 35 (*p* < 0.05, *p* < 0.001). And the liver NEFA levels of quails in the model group were extremely significantly increased (*p* < 0.001). See Table 7 and Table 8 and Figure 10 and Figure 11.

### 3.6. Enzyme Activities Related to Uric Acid and Lipid Metabolism in Model Animals

Compared with the normal group, the serum XOD activity of quails in the model group was significantly increased at 14, 21, and 35 days (*p* < 0.05, *p* < 0.001), with no significant difference at day 28 (*p >* 0.05). Compared with the normal group, the liver XOD activity of quails in the model group was extremely significantly increased (*p* < 0.001).

**Figure 12:**
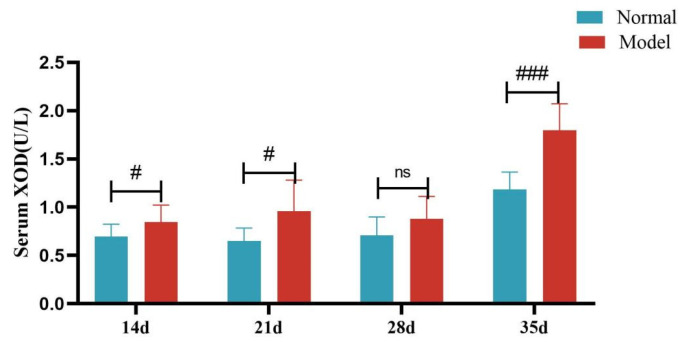
Serum XOD Activity of Quails at 7, 14, 21, 28, and 35 Days. (Note: Compared with the normal group, ^#^ *p* < 0.05,^###^ *p* < 0.001, ns indicates *p* > 0.05).

**Figure 13:**
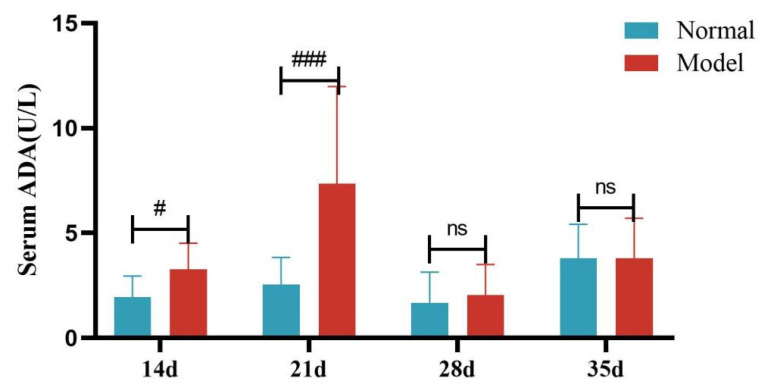
Serum ADA Activity of Quails at Days 7, 14, 21, 28, and 35. (Note: Compared with the normal group, ^#^ *p* < 0.05,^###^ *p* < 0.001, ns indicates *p* > 0.05).

**Figure 14:**
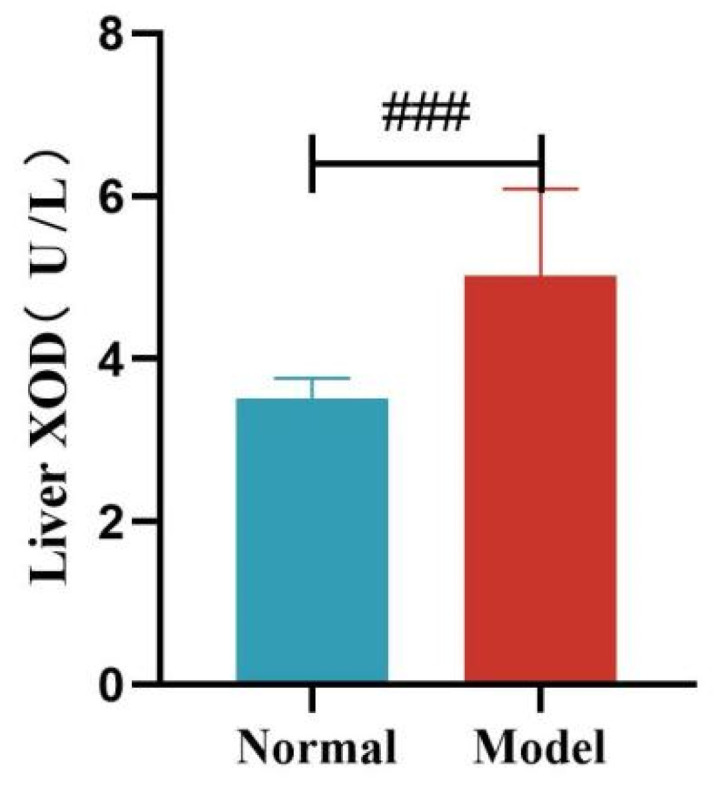
Liver XOD Activity of Quails. (Note: Compared with the normal group, ^###^ *p* < 0.001).

**Figure 15:**
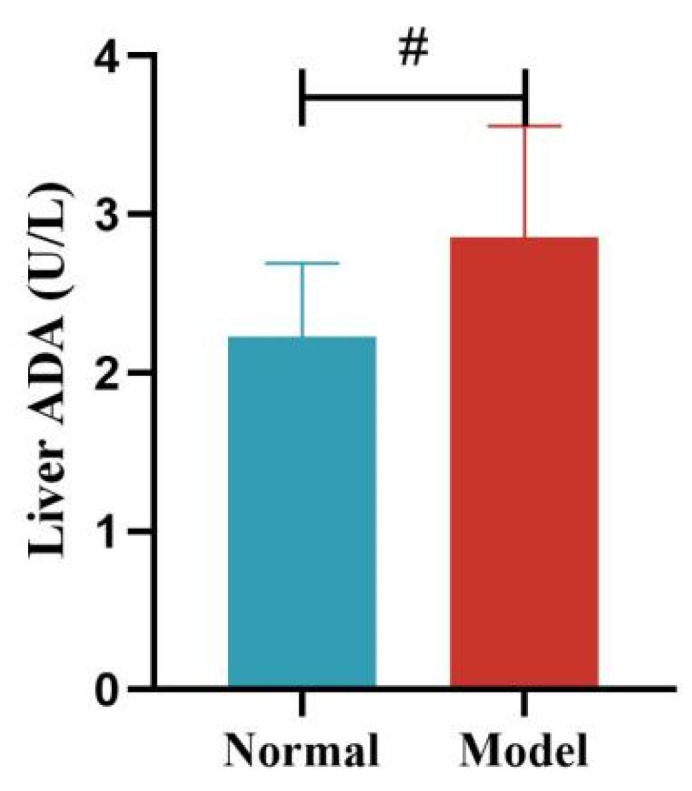
Liver ADA Activity of Quails. (Note: Compared with the normal group, ^#^ *p* < 0.05).

Compared with the normal group, the serum ADA activity of quails in the model group was significantly increased at days 14 and 21 (*p* < 0.05, *p* < 0.001), with no significant difference at days 28 and 35 (*p >* 0.05). Compared with the normal group, the liver ADA activity of quails in the model group was significantly increased (*p* < 0.05). See Table 9 and Table 10 and Figure 12, Figure 13, Figure 14 and Figure 15.

**Figure 16:**
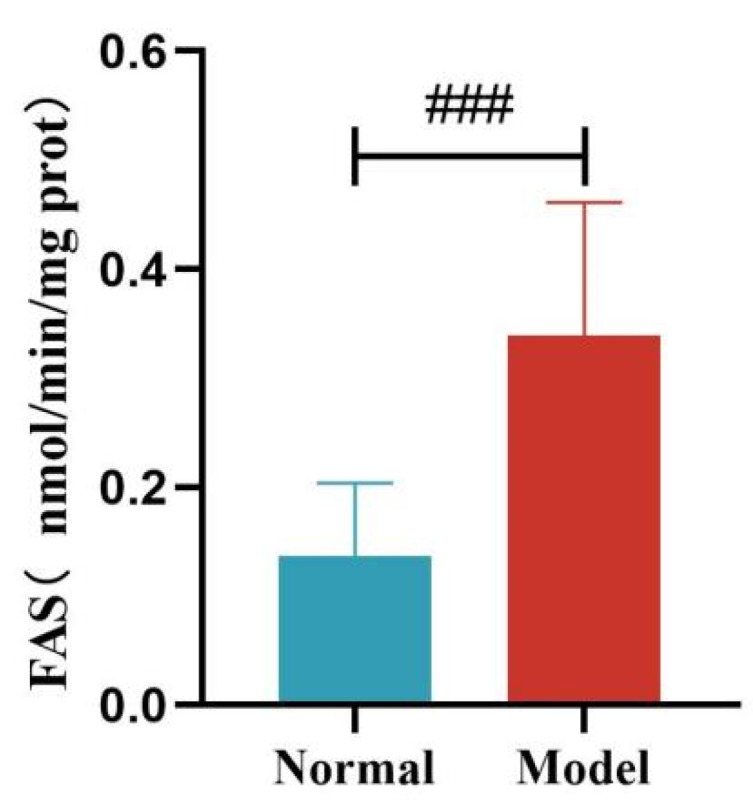
Liver FAS Levels of Quails. (Note: Compared with the normal group, ^###^ *p* < 0.001).

**Figure 17:**
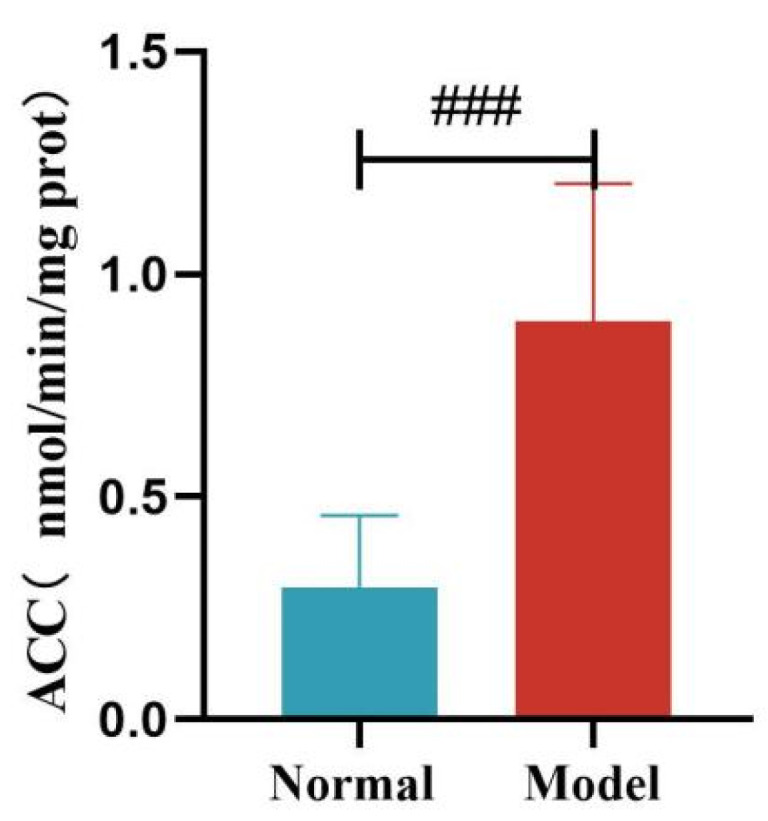
Liver ACC Levels of Quails. (Note: Compared with the normal group, ^###^ *p* < 0.001).

Compared with the normal group, the liver FAS and ACC activity of quails in the model group was significantly increased (*p* < 0.01, *p* < 0.001). See Table 11 and Figure 16 and Figure 17.

**Figure 18:**
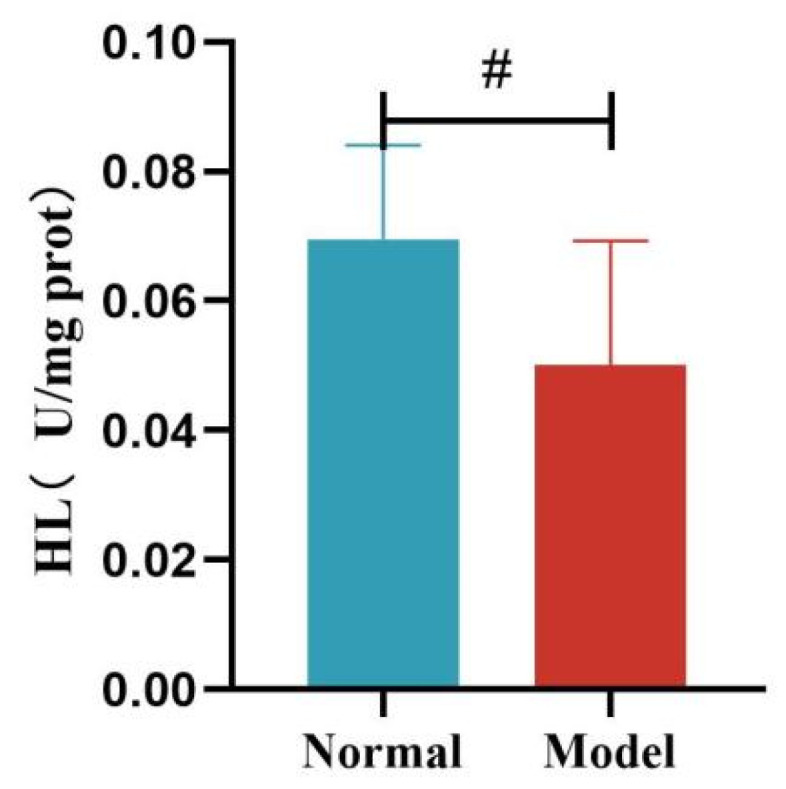
Liver HL Levels of Quails. (Note: Compared with the normal group, ^#^ *p* < 0.05.)

**Figure 19:**
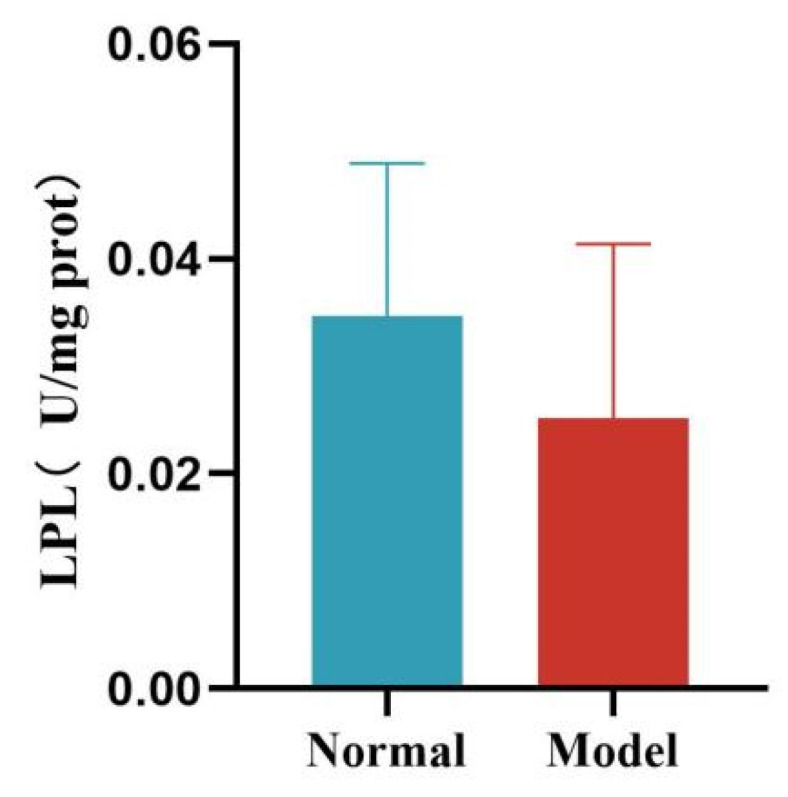
Liver LPL Levels of Quails.

Compared with the normal group, the liver HL activity of quails in the model group was significantly decreased (*p* < 0.05). Compared with the normal group, the liver LPL activity of quails in the model group was decreased, but the difference was not statistically significant (*p* > 0.05). See Table 12 and Figure 18 and Figure 19.

### 3.7. Liver Pathological Changes

**Figure 20:**
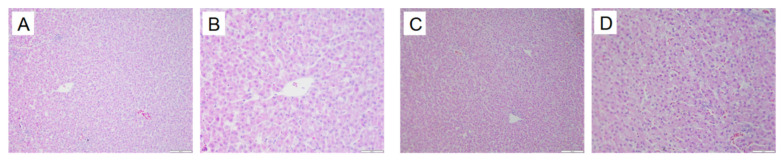
Liver pathological changes in Quails (200×, 400×) (size: 100 μm, 50 μm). Normal group 200× (**A**), Normal group 400× (**B**), Model group 200× (**B**), Model group 400× (**D**).

In the normal group, the hepatic lobule structure of quail livers is clear, with the central vein as the center, and the hepatic cords are neatly arranged in a radial pattern, with hepatocytes of uniform size. In the model group, the hepatic tissue structure of quails is normal, but some hepatocytes show fatty degeneration. See Figure 20.

### 3.8. Kidney Pathological Changes in Animals

**Figure 21:**
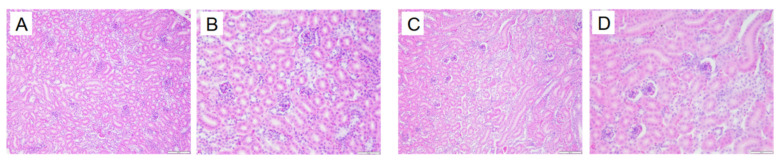
Kidney pathological changes in Quails (200×, 400×) (size: 100 μm, 50 μm). Normal group 200× (**A**), Normal group 400× (**B**), Model group 200× (**C**). Model group 400× (**D**).

In the normal group, the renal tissue structure is uniform, the boundaries of glomeruli and renal capsules are clear, and the renal tubule structure is intact. In the model group, the kidneys show glomerular atrophy, the cavity of the glomerular capsule becomes larger, and vacuolization occurs in the renal tubules. See Figure 21.

## 4. Discussion

The prevalence of hyperuricemia (HUA) among adults in China is 14.0%, making it the second most common metabolic disease in China after diabetes [2]. The overall prevalence of dyslipidemia is 35.6%, and this rate continues to increase each year [3]. Hyperlipidemia has already been regarded as an early warning sign for a range of health risks. Epidemiological data indicate that 60% of patients with hyperuricemia also have hyperlipidemia [6]. According to the 2019 Chinese Guidelines for the Diagnosis and Treatment of Hyperuricemia and Gout, 67% of patients with hyperuricemia and gout have combined lipid metabolism disorders [7]. Among patients with HUA, 75% to 80% present with hyperlipidemia, while 82% of patients with hyperlipidemia have hyperuricemia [8]. Hyperuricemia and hyperlipidemia often coexist, and elevated uric acid can aggravate lipid metabolism disorders [9]. This study conducted data analysis through NHANES and found that, there is a reciprocal relationship between uric acid and lipid metabolism, with the increase in uric acid level, the prevalence of hyperlipidemia increased, and the coexistence of hyperuricemia and hyperlipidemia greatly increases the metabolic burden and significantly raises the risk of various cardiovascular diseases. In the analysis of NHANES data, it was found that the age of HUA group was significantly lower than that of non-HUA group, suggesting that uric acid-related metabolic diseases showed a younger trend. Compared with the control group, the BMI and the risk of hypertension in the HUA group were significantly increased, suggesting that HUA is closely related to lipid metabolism and cardiovascular metabolic syndrome, and uric acid and lipid metabolism interact with each other. Therefore, the establishment of a stable and reliable HUA-HLP model is of great importance for in-depth study of the pathological mechanisms and drug development.

At present, animal models such as mice and rats are commonly used to simulate the clinical development of HUA-HLP, typically by administering chemical agents to inhibit uricase activity [15,16,17,18]. However, there is a significant difference between these modeling methods and the causes of the disease in clinical patients, which leads to certain limitations. Previous studies by our research group have found that a high-fat diet prepared with lard or a high-purine diet prepared with yeast extract powder can induce Defake quails to develop abdominal obesity, hyperuricemia, hyperlipidemia, and metabolic syndrome [19,20,21,22,23]. Quails are small in size, adapt well to the environment, and are easy to raise. The hyperuricemia quail model has been included in the National Human Disease Animal Model Resource Bank and has been rated as a Class B animal model for metabolic diseases by the Chinese Association for Laboratory Animal Sciences. Therefore, quails were chosen as the experimental animals in this study to simulate the etiology and course of disease in clinical patients.

In current studies on HUA-HLP animal models, some researchers [24] have established HUA-HLP rat models by oral gavage of a high-fat emulsion (composed of 25% lard, 10% cholesterol, 2% sucrose, 1% methimazole, 25% Tween-80, and 20% propylene glycol) combined with potassium oxonate (300 mg/kg) and adenine (20 mg/kg) for five weeks. Other researchers [25] have induced HLP and HUA rat models by feeding SD rats a high-fat diet (20% protein, 20% carbohydrate, 60% fat) in combination with oral administration of adenine and ethambutol for eight weeks. Previous studies by our research group [26,27,28,29] have found that, when Defake quails are fed a high-purine diet (15 g/kg yeast extract powder), hyperuricemia appears in the first week of modeling, and significant changes in TC and TG levels are observed by the third week. This modeling method also increases the abdominal fat rate and abdominal fat mass in quails, exhibiting the pathological feature of abdominal obesity [30]. When Defake quails were induced with a high-fat diet (14% lard, 1% cholesterol, 85% basic feed) for two weeks, hyperuricemia, hypertriglyceridemia, and abdominal obesity were observed. Other studies [30,31,32,33,34] have induced SD rats with 10% fructose water and found that TG levels significantly increased in the first week of modeling, and UA levels changed significantly by the third week, successfully inducing hypertriglyceridemia with HUA.

The main characteristic of HUA-HLP is the combination of uric acid metabolism disorder and lipid metabolism abnormality. The present study showed that, in the process of inducing the HUA-HLP model by a high-purine and high-fat diet, the modeling criteria were met as early as the first week. The model animals exhibited a significant increase in uric acid level, indicating the formation of hyperuricemia; at the same time, there were changes in blood lipid levels, mainly significant increases in serum TG and TC, which met the diagnostic criteria for hypertriglyceridemia and hypercholesterolemia. By the third week of high-purine and high-fat diet induction, HUA, hypertriglyceridemia, and hypercholesterolemia persisted, and significant changes in serum HDL-C and LDL-C levels were observed, presenting as hypo-HDL-cholesterolemia and hyper-LDL-cholesterolemia, which continued until the end of the experiment. In the early stage of model construction, uric acid metabolism disorder occurred first, while the main feature of lipid metabolism disorder was hypertriglyceridemia and hypercholesterolemia; in the middle stage of model construction, hypo-HDL-cholesterolemia and hyper-LDL-cholesterolemia appeared; at the late stage of model construction (before the quails were sacrificed), serum UA, TG, TC, HDL-C, and LDL-C all showed significant differences, indicating that uric acid and lipid metabolism had become severely disordered.

During the induction period with a high-purine and high-fat diet, the activities of purine metabolism enzymes XOD and ADA were significantly increased, indicating that the uric acid synthesis pathway was activated [35,36]. At the same time, the activities of key enzymes in fatty acid synthesis, ACC and FAS, were significantly upregulated, while the activities of lipoprotein metabolic enzymes LPL and HL were decreased. This suggests that there was both enhanced lipid synthesis [37,38,39,40,41] and inhibited lipid breakdown in the quails [42,43,44,45], resulting in severe disruption of lipid homeostasis.

In summary, this study successfully established a HUA-HLP model by inducing Defake quails with a high-purine and high-fat diet. On this basis, the pathological features of the model were systematically explored, and changes in the activities of key enzymes involved in purine synthesis as well as in lipid synthesis and degradation were observed. The aim was to provide a more comprehensive investigation into the pathological states of hyperuricemia and hyperlipidemia, and to offer new ideas for constructing experimental models that reflect the dietary imbalance seen in clinical patients and the disease course of uric acid and lipid metabolism disorders.

## 5. Conclusions

This study successfully established a HUA-HLP model by inducing Defake quails with a high-purine and high-fat diet. On this basis, the pathological features of the model were systematically explored, and changes in the activities of key enzymes involved in purine synthesis as well as in lipid synthesis and degradation were observed. The aim was to provide a more comprehensive investigation into the pathological states of hyperuricemia and hyperlipidemia, and to offer new ideas for constructing experimental models that reflect the dietary imbalance seen in clinical patients and the disease course of uric acid and lipid metabolism disorders.

## Figures and Tables

**Table 1:** Weighted baseline characteristics of included participants.

Variable	Overall	Non-Hyperuricemia	Hyperuricemia	*p* Value
	(*n* = 2172)	(*n* = 1739)	(*n* = 379)	
Sociodemographic characteristics				
Age (years)	48.97 ± 6.69	53.42 ± 6.49	42.76 ± 6.54	<0.001
Sex (%)				0.342
Female	1130 (53.4)	962 (53.0)	168 (56.6)	
Male	1042 (46.6)	831 (47.0)	211 (43.4)	
Race (%)				<0.001
Mexican American	210 (3.4)	128 (2.7)	52 (9.4)	
Non-Hispanic Black	590 (7.9)	370 (6.6)	122 (19.5)	
Non-Hispanic White	1257 (80.0)	1001 (82.9)	124 (55.2)	
Other Hispanic	244 (3.7)	133 (2.7)	66 (12.5)	
Other Race	203 (4.9)	161 (5.1)	15 (3.5)	
Education level (%)				<0.001
Below high school	490 (14.6)	286 (11.3)	204 (42.8)	
High school	515 (22.1)	424 (21.2)	91 (29.9)	
Above high school	1167 (63.3)	1083 (67.5)	84 (27.2)	
Marital status (%)				<0.001
Married	1271 (66.4)	1080 (68.2)	191 (50.6)	
Unmarried	901 (33.6)	713 (31.8)	188 (49.4)	
Poverty-income ratio (%)				
<1.3	603 (16.5)	419 (14.1)	184 (38.1)	<0.001
1.3–3.5	858 (38.8)	715 (38.2)	143 (43.7)	
>3.5	711 (44.7)	659 (47.7)	52 (18.2)	
Behavioral characteristics				
Smoking status (%)	1111 (49.6)	927 (49.9)	184 (46.6)	0.337
Yes	1061 (50.4)	866 (50.1)	195 (53.4)	
No				
Alcohol consumption (%)	1510 (73.4)	1272 (75.4)	238 (55.8)	<0.001
Yes	662 (26.6)	521 (24.6)	141 (44.2)	
No				
Physical activity (%)				<0.001
Yes	938 (46.5)	824 (48.3)	114 (31.4)	
No	1234 (53.5)	969 (51.7)	265 (68.6)	
Health characteristics				
Body mass index (kg/m^2^)				0.276
<25	569 (26.6)	455 (25.8)	114 (33.5)	
25–30	760 (36.2)	638 (36.7)	122 (31.9)	
≥30	843 (37.2)	700 (37.5)	143 (34.6)	
Diabetes (%)				<0.001
Yes	540 (19.3)	398 (17.5)	142 (35.6)	
No	1632 (80.7)	1395 (82.5)	237 (64.4)	
Hypertension (%)				0.001
Yes	1345 (58.7)	1074 (57.3)	271 (70.3)	
No	827 (41.3)	719 (42.7)	108 (29.7)	
Hyperlipidemia	−0.06 [−2.07, 2.11]	0.20 [−1.89, 2.35]	−1.88 [−3.32, 0.44]	<0.001

**Table 2:** Multivariate logistic regression analysis results.

	Model1		Model2		Model3	
	OR (95%CI)	*p* Value	OR (95%CI)	*p* Value	OR (95%CI)	*p* Value
Hyperlipidemia (Continuous)	0.83 (0.78, 0.89)	<0.001	0.85 (0.79, 0.92)	<0.001	0.89 (0.82, 0.96)	0.004
Hyperlipidemia (Categorical)						
Q1	Ref		Ref		Ref	
Q2	0.46 (0.33, 0.66)	<0.001	0.44 (0.27, 0.72)	0.002	0.53 (0.31, 0.92)	0.027
Q3	0.36 (0.24, 0.55)	<0.001	0.38 (0.25, 0.59)	<0.001	0.52 (0.32, 0.82)	0.009
Q4	0.21 (0.13, 0.35)	<0.001	0.23 (0.13, 0.41)	<0.001	0.31 (0.17, 0.58)	0.002
p for trend		<0.001		<0.001		0.001

**Table 3:** Changes in Body Weight of Quails at Different Time Points (g, $\bar{x}$ ± s).

Time Point	Normal Group	Model Group
0 day	148.53 ± 10.73	158.53 ± 12.85
5 day	161.49 ± 13.36	162.60 ± 6.16
10 day	157.57 ± 17.47	163.71 ± 8.51
15 day	167.53 ± 17.84	187.06 ± 9.78 ^##^
20 day	140.13 ± 14.12	174.40 ± 10.23 ^###^
25 day	169.11 ± 13.70	195.67 ± 13.39 ^###^
30 day	176.03 ± 11.60	196.82 ± 12.47 ^###^
35 day	198.50 ± 10.69	222.02 ± 12.93 ^###^

Note: Compared with the normal group, ^##^ *p* < 0.01, ^###^ *p* < 0.001.

**Table 4:** Changes in Food Intake of Quails at Different Time Points (g, $\bar{x}$ ± s).

Time Point	Normal Group	Model Group
0 day	217.92 ± 16.44	199.96 ± 0.04
5 day	238.07 ± 12.07	240.00 ± 14.14
10 day	251.94 ± 8.37	255.99 ± 5.47
15 day	293.17 ± 57.62	293.33 ± 57.74
20 day	409.73 ± 0.31	409.45 ± 0.03
25 day	414.47 ± 10.02	415.24 ± 12.79
30 day	428.99 ± 0.48	429.87 ± 0.11
35 day	429.63 ± 0.06	429.45 ± 0.04

**Table 5:** Serum and Fecal Uric Acid Levels of Quails at 7, 14, 21, 28, and 35 Days (μmol/L, $\bar{x}$ ± s).

	Serum UA Level (μmol/L)	Fecal and Urinary Mixed UA Level (μmol/L)
7d Normal (*n* = 10)	89.20 ± 23.22	454.52 ± 77.09
7d Model (*n* = 10)	284.07 ± 105.16 ^###^	552.25 ± 81.01 ^#^
14d Normal (*n* = 10)	84.01 ± 28.78	599.66 ± 72.08
14d Model (*n* = 10)	405.35 ± 52.39 ^###^	758.46 ± 80.40 ^###^
21d Normal (*n* = 10)	179.09 ± 25.88	218.35 ± 35.06
21d Model (*n* = 10)	331.78 ± 40.15 ^###^	346.86 ± 90.01 ^###^
28d Normal (*n* = 10)	161.68 ± 37.52	340.09 ± 54.00
28d Model (*n* = 10)	305.05 ± 47.43 ^###^	649.89 ± 53.19 ^###^
35d Normal (*n* = 10)	183.12 ± 30.00	137.64 ± 27.69
35d Model (*n* = 10)	248.55 ± 22.05 ^###^	214.67 ± 74.14 ^##^

Note: Compared with the normal group, ^#^ *p* < 0.05, ^##^ *p* < 0.01, ^###^ *p* < 0.001.

**Table 6:** Serum TC/TG/LDL-C/HDL-C of Quails at Days 7, 14, 21, 28, and 35 (mmol/L, $\bar{x}$ ± s).

	TG	TCHO	LDL-C	HDL-C
7d Normal (*n* = 10)	0.55 ± 0.28	5.69 ± 0.79	9.22 ± 3.55	5.52 ± 2.59
7d Model (*n* = 10)	1.19 ± 0.32 ^###^	7.96 ± 0.80 ^###^	12.30 ± 1.68 ^#^	5.98 ± 1.26
14d Normal (*n* = 10)	0.50 ± 0.19	9.87 ± 1.85	5.26 ± 1.62	8.26 ± 2.77
14d Model (*n* = 10)	0.88 ± 0.20 ^###^	13.26 ± 2.48 ^##^	12.00 ± 2.43 ^###^	6.74 ± 1.28
21d Normal (*n* = 10)	0.91 ± 0.13	7.82 ± 1.71	4.27 ± 0.69	13.63 ± 2.28
21d Model (*n* = 10)	1.25 ± 0.38 ^###^	15.07 ± 2.49 ^###^	6.83 ± 1.56 ^###^	10.95 ± 1.56 ^###^
28d Normal (*n* = 10)	0.67 ± 0.17	6.74 ± 0.80	4.79 ± 0.63	10.04 ± 1.17
28d Model (*n* = 10)	1.25 ± 0.28 ^###^	9.22 ± 1.36 ^###^	6.85 ± 1.87 ^###^	8.25 ± 0.53 ^##^
35d Normal (*n* = 10)	0.72 ± 0.20	1.38 ± 0.49	0.79 ± 0.39	4.95 ± 0.93
35d Model (*n* = 10)	1.17 ± 0.13 ^###^	3.09 ± 0.89 ^###^	1.77 ± 0.57 ^###^	3.84 ± 1.35 ^#^

Note: Compared with the normal group, ^#^ *p* < 0.05, ^##^ *p* < 0.01, ^###^ *p* < 0.001.

**Table 7:** Serum NEFA Levels of Quails at Days 14, 21, 28, and 35 (mmol/L, $\bar{x}$ ± s).

Group	Serum NEFA (mmol/L)
14d Normal (*n* = 10)	0.43 ± 0.199
14d Model (*n* = 10)	0.800 ± 0.19 ^###^
21d Normal (*n* = 10)	0.63 ± 0.13
21d Model (*n* = 10)	1.32 ± 0.39 ^###^
28d Normal (*n* = 10)	0.35 ± 0.08
28dModel (*n* = 10)	0.44 ± 0.09 ^#^
35d Normal (*n* = 10)	0.17 ± 0.11
35d Model (*n* = 10)	0.49 ± 0.26 ^##^

Note: Compared with the normal group, ^#^ *p* < 0.05, ^##^ *p* < 0.01, ^###^ *p* < 0.001.

**Table 8:** Liver NEFA Levels of Quails (mmol/L, $\bar{x}$ ± s).

Group	Liver NEFA (mmol/L)
Normal (*n* = 10)	0.006 ± 0.003
Model (*n* = 10)	0.021 ± 0.005 ^###^

Note: Compared with the normal group, ^###^ *p* < 0.001.

**Table 9:** Serum XOD and ADA Activity of Quails at Days 14, 21, 28, and 35 (U/L, $\bar{x}$ ± s).

	Serum XOD (U/L)	Serum ADA (U/L)
14d Normal (*n* = 10)	0.69 ± 0.13	1.94 ± 1.01
14d Model (*n* = 10)	0.85 ± 0.18 ^#^	3.28 ± 1.25 ^#^
21d Normal (*n* = 10)	0.65 ± 0.14	2.55 ± 1.29
21d Model (*n* = 10)	0.96 ± 0.32 ^#^	7.40 ± 4.64 ^###^
28d Normal (*n* = 10)	0.71 ± 0.19	1.68 ± 1.47
28d Model (*n* = 10)	0.88 ± 0.23	2.04 ± 1.46
35d Normal (*n* = 10)	1.19 ± 0.18	3.81 ± 1.61
35d Model (*n* = 10)	1.80 ± 0.28 ^###^	3.81 ± 1.91

Note: Compared with the normal group, ^#^ *p* < 0.05, ^###^ *p* < 0.001.

**Table 10:** Liver XOD and ADA Activity of Quails (U/L, $\bar{x}$ ± s).

	Liver XOD (U/L)	Liver ADA (U/L)
Normal (*n* = 10)	3.51 ± 0.25	2.23 ± 0.46
Model (*n* = 10)	5.02 ± 1.07 ^###^	2.86 ± 0.70 ^#^

Note: Compared with the normal group, ^#^ *p* < 0.05, ^###^ *p* < 0.001.

**Table 11:** Liver FAS and ACC Activity of Quails (nmol/min/mg prot, $\bar{x}$ ± s).

Group	FAS	ACC
Normal (*n* = 10)	0.14 ± 0.06	0.30 ± 0.16
Model (*n* = 10)	0.34 ± 0.12 ^##^	0.89 ± 0.31 ^###^

Note: Compared with the normal group, ^##^ *p* < 0.01, ^###^ *p* < 0.001.

**Table 12:** Liver HL and LPL Activity of Quails (U/mg prot, $\bar{x}$ ± s).

Group	HL	LPL
Normal (*n* = 8)	0.070 ± 0.015	0.035 ± 0.014
Model (*n* = 8)	0.050 ± 0.019 ^#^	0.025 ± 0.016

Note: Compared with the normal group, ^#^ *p* < 0.05.

## Data Availability

The datasets used and/or analyzed during the current study are available from the corresponding author on reasonable request. All data generated or analyzed during this study are included in this published article.
